# Artificially intelligent modeling of macromolecular assemblies

**DOI:** 10.3389/fmolb.2026.1856649

**Published:** 2026-10-01

**Authors:** Margherita Bini, Valentina Tozzini

**Affiliations:** 1 Scuola Normale Superiore, Pisa, Italy; 2 Istituto Nanoscienze, National Research Council (CNR-NANO), NEST-SNS, Pisa, Italy; 3 INFN Sez Pisa, Largo Bruno Pontecorvo Pisa, Pisa, Italy

**Keywords:** AI-based modeling, amyloid aggregation, coarse-grained models for proteins, FF parameterization, intrinsically disordered proteins, protein interactions

## Abstract

The study of macromolecular assembly formation is one of the most challenging tasks in computational modeling as it requires extensive exploration of conformational and configurational space, especially when driven by conformational transitions or folding instabilities, which are typically associated with high energy barriers. Although this type of study has traditionally been addressed *in silico* by using techniques that reduce the computational cost of simulations, such as coarse-grained models, artificial intelligence (AI) has recently entered the field at multiple levels, aiding in parameter optimization or trajectory analysis and even replacing potentials or force calculations. Recently developed algorithms such as AlphaFold (AF) and RoseTTAFold enable the direct prediction of final structures with high efficiency and confidence, an achievement that earned them the 2024 Nobel Prize in Chemistry. In this study, we review and analyze the different AI-based strategies used to model protein structure and dynamics and simulate macromolecular assemblies, comparing them with traditional methods, to highlight their respective advantages and limitations. By placing these approaches along an ideal continuum in which accuracy increases as interpretability decreases, we show that hybrid solutions and combinations of methods can be devised to maximize their strengths. Finally, we perspectively outline practical strategies for implementing such integrated approaches in a coherent manner, preserving accuracy and feasibility while maintaining insight into aggregation pathways, intermediate structures, and the molecular mechanisms underlying the phenomenon.

## 1 Introduction

Protein aggregation is ubiquitously involved in a large number of cell processes, such as the regulation of metabolism, the assembly of cell machinery, the formation of membraneless organelles and internal cell structures (Banani et al., 2017), and the functioning of artificial molecular sensors (Montepietra et al., 2025). Therefore, great efforts in molecular modeling are aimed at disclosing the interface structure of the proteins and the determinants of their formation, generally driven by a complex interplay between shape complementarity, electrostatics, and hydrophobicity (Keskin et al., 2008; Navarro and Ventura, 2022). The case of intrinsically disordered proteins (IDPs), which are most often involved in diseases due to dysfunctional aggregation (Uversky, 2017), is even more challenging because of the additional degrees of freedom (DoFs) introduced by their variable conformations.

Atomistic force field (FF)-based molecular dynamics (MD) is a powerful tool for these studies, offering a unique mechanistic view of the specific driving forces of aggregation and unraveling interface formation pathways and intermediate states that can be exploited for drug design or for identifying possible mutations capable of interfering with the process (Österlund et al., 2020). However, aggregation extends to macroscopic scales that remain marginally accessible *in silico* using fully atomistic representations (Palermo et al., 2020). Several shortcuts routes were followed to overcome this obstacle, which usually involve enhancing the conformational sampling (Leman et al., 2020) and using simplified (i.e., low resolution or coarse-grained (CG) (Heo et al., 2022)) representation of the system. The leap of artificial intelligence (AI) into this field has boosted all of these aspects (Tozzini and Giulivi, 2025): it can be used to optimize the conformational search (Noé et al., 2019), parameterize the CG force fields, or even directly predict forces and trajectories, as well as contact interfaces and structures, as in RoseTTafold (Humphreys et al., 2021) or the latest versions of AlphaFold (AF) (Jumper et al., 2021).

All of these tasks have in common that they are data-driven (Figure 1a). On general grounds, machine learning (ML) methods can help whenever large amounts of data must be distilled into few informative parameters, a task that properly trained (deep) neural network (NN) solve excellently. To model the aggregation with CG models, one generally first parameterizes the interactions of the CG force field (CG-FF) (Figure 1b) using datasets of different kinds (Figure 1a), then calculates energies and forces, and produces trajectories (Figure 1c), finally leading to folding or aggregation (Figure 1d). ML can enter the process at several levels (Figure 1e). It can be used to optimize the parameterization of the FF or train NNs to predict energies, forces, or even trajectories. The different approaches may differ in the architecture of the network, in the specific targeted quantities, and in the amount and type of learned features, which influence the transferability and accuracy of the model, as well as the cost of learning and interpretability of results.

**FIGURE 1:**
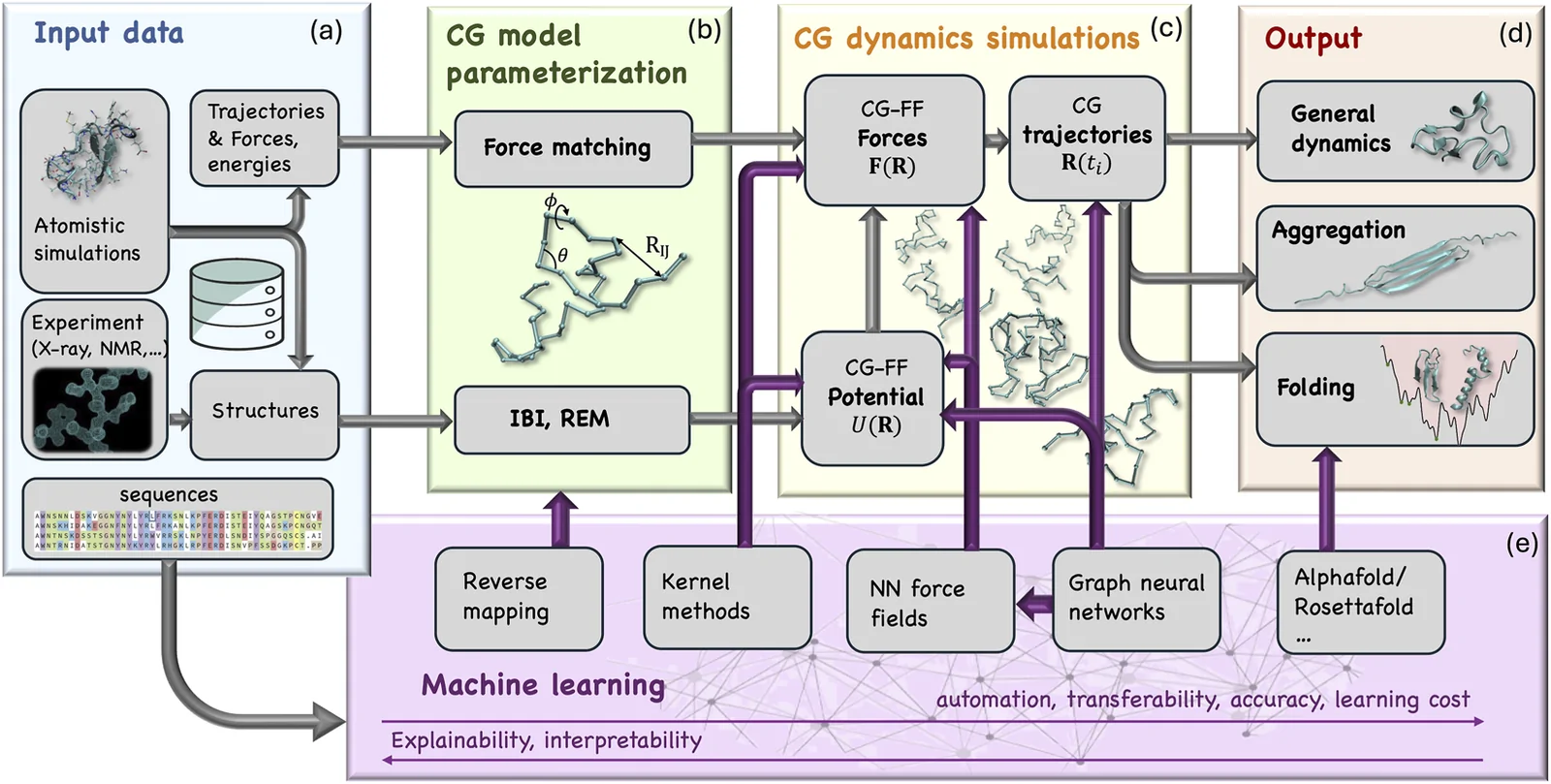
Scheme for a CG model building and its use to study dynamics, aggregation or folding. The top boxes from lef to right show the different phases of data driven CG model building and parameterization (blue **(a)** and green **(b)** boxes) generation of dynamics trajectories and output production and analysis (yellow **(c)** and orange **(d)** boxes). On the bottom, in the pink box **(e)**, different ML based algorithms are reported, with the arrows indicating the level at which they can act in the workflow.

In this study, we review the most popular methods (regression, kernel methods, NN-FFs, graph neural network (GNN), and some variants or combinations), placing them along a continuum in which accuracy and transferability increase as physical interpretability decreases (Figure 1e), highlight their advantages and limitations, and find complementary strengths especially with respect to the study of protein aggregation. Among other things, we specifically discuss how analytical FFs, which are placed at one end of the continuum and offer high physical interpretability but limited accuracy, can be parameterized using ML regression methods to optimize intra-peptide potential parameters, thereby accurately representing the flexibility and conformational changes affecting disordered proteins (Greener, 2024); how NN-FF and Kernel methods can capture many-body effects in CG model interactions (Wang J. et al., 2020); and how graph neural networks can be physically informed to combine accuracy, efficiency, and interpretability (Sharma and Fink, 2026). In the final part, we focus on the algorithms for the direct prediction of proteins and assembly structures, specifically AlphaFold, which represents the extreme of the continuum, where accuracy is maximal but interpretability is minimal; we report how side-output from AlphaFold can be strategically used to inform CG models, simultaneously providing a possible route to improve their interpretability (Bini et al., 2025), as an example of combined use of AI-based and conventional methods for protein assembly modeling.

In order to define key concept and a technical background to address this discussion, we first step back and review the more conventional approaches to build and parameterize the CG models. This will also highlight their limitations and how they can be overcome by means of AI methods.

## 2 Coarse-grained models and force fields

In CG models, atoms are grouped in “beads” whose interactions include high levels of empiricism. The mapping specificity (resolution, i.e., amount of atoms in the bead and where to locate the bead with respect to them) influences the quality of the model, and the optimal choice is still a matter of debate (Foley et al., 2020). Here, we assume the mapping as given. Even so, other choices must be made on the representation of interactions and the strategy to evaluate them, which are described in the following subsections.

### 2.1 Atomistic to coarse-grained mapping, entropy change, and potential of mean force

The atomistic-to-CG mapping scheme (Jin et al., 2022) can generally be regarded as a function $\left\{R_{I}\right\}=M_{I}\left(\left\{r_{i}\right\}\right)$ , with $r_{i}$ (i=1,…,n) denoting the atomistic coordinates and $R_{I}$ ( I=1,…,N , N<n ) the CG coordinates; the mapping is generally linear if both set of coordinates, collectively denoted by **r** and **R**, are Cartesian. The model computational cost, accuracy, predictive power, and other features depend on the mapping function. For the case of study of protein aggregation, a good choice is the single bead per residue resolution, which is the minimal choice allowing the description of the protein internal structure and the sequence specificity of the interface (Tozzini, 2010). The additional choice of placing the CG bead of a specific atom, usually the C α , is also known as “decimation mapping” (Giulini et al., 2020).

The mapping operation is associated to an entropy change. It is useful to define the degeneracy associated to a CG configuration Ω(R)=∫drδ(R−M(r)) , where M is the collective mapping function whose I th component is $M_{I}$ , i.e., $M\left(\left\{r_{i}\right\}\right)=\left(M_{1}\left(\left\{r_{i}\right\}\right),\ldots ,M_{N}\left(\left\{r_{i}\right\}\right)\right)$ . We also define the average operation $\bar{f}\left(R\right)={\left(\Omega \left(R\right)\right)}^{-1}\int drf\left(r\right)\delta \left(R-M\left(r\right)\right)$ over the atomistic configurations mapped onto a given CG configuration R . Since Ω has dimension of ${\text{volume}}^{n-N}$ , where n is the number of atoms and N is that of CG beads, the numerical degeneracy (number of atomistic configurations corresponding to a CG configuration R ) is rather $\Omega \left(R\right)/v^{n-N}$ , where v is a unit volume of the configurational space^1^. By applying the average operation to the atomistic canonical probability distribution p , using the atomistic canonical partition function $z=\int dr\;e^{-\beta u\left(r\right)}$ , we obtain Equation 1:

$$\bar{p}\left(R\right)=\frac{\bar{e^{-\beta u\left(r\right)}}}{z}\left(R\right)=\frac{1}{z}\frac{\int dr\;e^{-\beta u\left(r\right)}\;\delta \left(M\left(r\right)-R\right)}{\Omega \left(R\right)}≳\frac{e^{-\beta \bar{u}\left(R\right)}}{z}=p\left(\bar{u}\left(R\right)\right),$$ (1)

with β=1/kT , and the ≳ stems from applying the Jensen inequality to the Boltzmann factor^2^. This suggests that the probability of the CG state R , obtained by averaging the Boltzmann weights of all corresponding microstates, is always greater than or equal to the probability computed from the mean energy $\bar{u}$ alone, due to the convexity of the exponential function. The equality holds if the potential u(r) is uniform within the set of atomistic configurations that maps to **R**.

The CG probability distribution P(R) is obtained by marginalizing the atomistic probability p(r) over the atomistic configurations that map to R , as shown in Equation 2;

$$P\left(R\right)=\int dr\;p\left(r\right)\;\delta \left(M\left(r\right)-R\right)=\Omega \left(R\right)\bar{p}\left(R\right)\;\Rightarrow \;P\left(R\right)≳\Omega \left(R\right)p\left(\bar{u}\left(R\right)\right).$$ (2)

Inequality holds as for Equation 1. The atomistic-to-CG mapping involves a loss of entropy, due to the reduction in the number of degrees of freedom. This loss can be quantified as the Gibbs entropy difference between the atomistic and CG representations, as presented in Equation 3:

$$\frac{\Delta S}{k_{B}}=-\int dR\;P\left(R\right)\left[\ln\left(v^{N}P\left(R\right)\right)\right]+\int dr\;p\left(r\right)\left[\ln\left(v^{n}p\left(r\right)\right)\right]=\left\langle \ln\left(\frac{p\left(r\right)}{\bar{p}\left(R\left(r\right)\right)}\right)\right\rangle -\left\langle \ln\left(\frac{\Omega \left(R\left(r\right)\right)}{v^{n-N}}\right)\right\rangle ,$$ (3)

where ⟨⟩ is the statistical average on p . ΔS has two contributions: the first (hereafter $S_{\mathrm{rel}}$ ) is the Kullback–Liebner divergence (Kullback and Leibler, 1951) measuring the difference between the original p and its averaged version $\bar{p}$ , as shown in Equation 4:

$$S_{\mathrm{rel}}=k_{B}\left\langle \ln\left(\frac{p\left(r\right)}{\bar{p}\left(R\left(r\right)\right)}\right)\right\rangle =k_{B}\left\langle \ln\left(p\left(r\right)\right)\right\rangle -k_{B}\left\langle \ln\left(\bar{p}\left(R\left(r\right)\right)\right)\right\rangle ≲-\frac{1}{T}\left\langle u\left(r\right)\right\rangle +\frac{1}{T}\left\langle \bar{u}\left(R\left(r\right)\right)\right\rangle ,$$ (4)

and the last inequality turns to equality under the same conditions as of Equation 1. A trivial case is when u=const over all the atomistic configurations, in which case $u\left(r\right)=\bar{u}\left(R\right)=u$ and $S_{\mathrm{rel}}=0$ . The second term of Equation 3, here named $S_{\mathrm{map}}$ ^3^, depends only on the mapping function. For instance, for uniform mapping, i.e., when $\Omega =const=V^{n-N}$ ( V is the system volume), one has $S_{\mathrm{map}}=-k_{B}\left(n-N\right)\ln\left(V/v\right)$ , i.e., $S_{\mathrm{map}}$ is negative and measures the reduction in DoFs. Non-uniform Ω is obtained, e.g., for non-linear mapping or beads of different composition/size, and still using Jensen inequality, it can be shown that in this case, one has $S_{\mathrm{map}}\geq -k_{B}\ln\left(\left\langle \Omega \left(R\right)\right\rangle /v^{n-N}\right)$ ^4^. We remark the different meaning of the two terms: $S_{\mathrm{rel}}$ measures the incapability of reproducing correctly all the atomistic features of the potential in CG representation, and $S_{\mathrm{map}}$ is due to reduction in DoFs. The entropy change ΔS may also be interpreted in light of the topological theory of phase transitions (Gori et al., 2022): a large ΔS signals that the atomistic-to-CG mapping may alter the topology of configuration space, which must be taken into account in the analysis of the conformational transitions with the CG model.

Given the CG probability defined in Equation 2, one can define a many-bead potential of mean force (PMF) U(R) , as shown in Equation 5:

$$\begin{array}{c} P\left(R\right)=\frac{e^{-\beta U\left(R\right)}}{Z},\;Z=\int dR\;e^{-\beta U\left(R\right)}\;\Rightarrow \;\\ \;\Rightarrow U\left(R\right)=-k_{B}T\ln\left(\bar{e^{-\beta u\left(R\left(r\right)\right)}}\right)\\ \;-k_{B}T\ln\left(\frac{\Omega \left(R\right)}{v^{n-N}}\right)-k_{B}T\ln\left(\frac{Z}{z}v^{n-N}\right). \end{array}$$ (5)

Therefore, U(R) is approximated—up to a constant—by the average of the energy of the micro-states mapping onto the CG configuration R plus an entropic term proportional to the logarithmic numerosity of those microstates: more degenerated CG states are entropically stabilized. This highlights the statistical content of the PMF U(R) , which is one of the reasons behind the difficulty of its evaluation.

### 2.2 Parametric CG interaction potentials

The many-body U(R) defined in Equation 5 reproduces the CG probability distribution P(R) defined in Equation 2. Targeting it directly to model a many-body $U^{CG}\left(R\right)$ is a possible strategy, made more accessible using AI-based methods described in the next section. In this section, we examine the more conventional strategies consisting in approximating it with analytical forms $U^{CG}\left(R,\gamma \right)$ , depending on a set of parameters {γ} . In the definition of the functional form, one can follow two diverging strategies: (i) preserving as much as possible the many-body nature of the potential, which requires very complex functions and a large number of parameters; or (ii) using simplified, easier-to-handle functional forms. Strategy (i) generally results in more transferable and accurate potentials, but the parameter optimization is more complex and the use in simulations is more expensive. On the other hand, strategy (ii) returns cheaper potentials easier to parameterize, cheaper to use in simulations, and with more direct physical interpretation, but generally less transferable and accurate.

In any case, $U^{CG}\left(R\right)$ can be associated to a CG probability distribution $P^{CG}\left(R\right)\propto e^{-\beta U^{CG}\left(R\right)}$ , and a relative entropy (Giulini et al., 2020) can be defined in Equation 6:

$$S_{\text{rel}}^{CG}=k_{B}\int dr\;p\left(r\right)\ln\left(\frac{p\left(r\right)\Omega \left(R\left(r\right)\right)}{P^{CG}\left(R\left(r\right)\right)}\right)=S_{\mathrm{rel}}+k_{B}\int dr\;p\left(r\right)\ln\left(\frac{\bar{p}\left(M\left(r\right)\right)\;\Omega \left(R\left(r\right)\right)}{P^{CG}\left(R\left(r\right)\right)}\right),$$ (6)

which returns $S_{\mathrm{rel}}$ when $P^{CG}\left(R\right)\equiv P\left(R\right)$ and is otherwise larger. Therefore, $S_{\mathrm{rel}}^{CG}$ is minimized by the CG potential that best represents the many-body PMF U(R) with the analytic $U^{CG}\left(R,\gamma \right)$ . One typical $U^{CG}$ functional form of type (ii) is completely separated in the following terms (Equation 7):

$$U^{CG}\left(Q;\gamma \right)=U_{\text{conf}}\left(\theta ,\phi ;\gamma_{\text{c}}\right)+U_{\text{hb}}\left(R_{IJ};\gamma_{\text{nb}}\right)+U_{\text{hy}}\left(R_{IJ};\gamma_{\text{hy}}\right)+U_{\text{el}}\left(R_{IJ};\gamma_{\text{el}}\right),$$ (7)

where Q is a set of internal variables (angles θ and dihedrals ϕ between subsequent ${\text{C}}_{\alpha }$ and relative distances $R_{IJ}$ ), and each potential term [configurational $U_{\text{c}\text{o}\text{n}\text{f}}$ , hydrogen bonding $U_{\text{h}\text{b}}$ , hydrophobic $U_{\text{h}\text{y}}$ , and electrostatic $U_{\text{e}\text{l}}$ (Bini et al., 2023)] has a direct physical interpretation. This form encompasses a vast class of models with different scopes and parameterization from the completely structure-biased Go-like models (Hoang and Cieplak, 2000), including those with increasing levels of transferability (Ghavami et al., 2012; Fenza et al., 2009; Mereghetti et al., 2016). In fact, the simple and transparent functional form allows easily tuning a residual level of structural bias at the intramolecular level to ensure, for example, the preservation of the correct fold of structured proteins (Trovato et al., 2013; Bini et al., 2025), or on the other way round, to reduce this bias when needed, e.g., to represent the conformational variability of disordered proteins (Bini et al., 2026; Tozzini et al., 2007; 2006; Alemani et al., 2009; Yap et al., 2008).

The extreme simplicity, manageability, and transparency of this functional form comes with a cost: it includes only up to the fourth body interactions (the dihedral term), and exact uncoupling between terms is assumed. Both of these are very strong approximations, which makes the accuracy of these models depend on the specific analytical form and on the specific strategy chosen to optimize the parameters. These are described in the following subsection and usually proceed through direct fits or iterative regression methods.

### 2.3 Parameter optimization strategies

The several methods available to optimize the set of parameters {γ} of $U^{CG}\left(Q;\gamma \right)$ rely on different target physical quantities. The relative entropy minimization (REM) targets the minimization of $S_{\mathrm{rel}}^{CG}$ through the vanishing of its partial derivatives with respect to parameters, as shown in Equation 8:

$$\frac{\partial S_{\text{rel}}^{CG}}{\partial \gamma_{i}}=k_{B}\left[\int drp\left(r\right)\frac{\partial U^{\text{CG}}}{\partial \gamma_{i}}-\int dRP^{CG}\left(R\right)\frac{\partial U^{\text{CG}}}{\partial \gamma_{i}}\right]\Rightarrow L_{\mathrm{REM,i}}={\left\langle \frac{\partial U_{\text{CG}}}{\partial \gamma_{i}}\right\rangle }_{\text{AA}}-{\left\langle \frac{\partial U_{\text{CG}}}{\partial \gamma_{i}}\right\rangle }_{\text{CG}}=0,$$ (8)

and automatically defines a vectorial loss function $L_{\mathrm{REM}}=\left\{L_{\mathrm{REM,i}}\right\}$ , whose components $L_{\mathrm{REM,i}}$ are the difference in $U_{CG}$ gradients with respect to parameters evaluated over CG and atomistic trajectories. The parameter space is explored moving in steps along the residual gradient direction, e.g., using the steepest descent algorithm (Chaimovich and Shell, 2011). This method requires repeated extensive CG simulations, making it increasingly expensive with the number of parameters and complexity of the potential.

The Boltzmann inversion (BI) method (Tschöp et al., 1998) takes advantage of the assumed separated form of $U^{CG}$ , leading to a decoupled form of $P^{CG}$ . If the total CG potential is a sum of independent terms, each depending on a single internal coordinate $Q_{i}$ , the total CG probability distribution factorizes into a product of independent marginal distributions, one for each coordinate, as presented in Equation 9:

$$U^{CG}\left(\left\{Q_{i}\right\},\gamma \right)=\underset{i}{\sum }U^{i}\left(Q_{i},\gamma \right)\;\Rightarrow \;P^{CG}=\underset{i}{\prod }P_{i}^{CG}\left(Q_{i},\gamma \right)=\underset{i}{\prod }\frac{e^{-\beta U^{i}\left(Q_{i},\gamma \right)}}{Z^{i}}\;Z^{i}=\int dQ_{i}\;e^{-\beta U^{i}\left(Q_{i},\gamma \right)}.$$ (9)

Each $P_{i}$ is approximated with the corresponding marginal distribution evaluated on the atomistic dataset $P_{i}^{CG}\left(Q_{i},\gamma \right)=P_{i}\left(Q_{i}\right)=\bar{p}\left(Q_{i}\right)\Omega \left(Q_{i}\right)$ . Inverting this relation via the Boltzmann factor yields each potential term: $U_{i}\left(Q_{i},\gamma \right)=-k_{B}T\ln\left(P_{i}\left(Q_{i}\right)\right)+const$ , i.e., it is assumed that Equation 2 holds for all CG internal coordinates separately. In practice, however, the marginalization of the single variables $Q_{i}$ implies the integration over others, which includes their coupling, although it was assumed null, intrinsically frustrating the procedure. The numerical solution of the set of equations of iterative BI (IBI) proceeds iteratively correcting the potentials with terms depending on the logarithmic difference in P and $P^{CG}$ , the latter evaluated from a CG simulations at each iteration. This defines the IBI, with variants differing in the strategy for updating the parameters (Mereghetti et al., 2016). As REM, this method targets the thermodynamic consistency of CG and atomistic systems and, in principle, leads to the best approximation of the many-body $U^{CG}$ with the given decoupled form, but the neglected correlations often produce numerical instability and slow convergence (Wang H. et al., 2020).

The problem is mitigated reintroducing partial correlations with more complex functional forms, e.g., a combined angular-dihedral term as in Equation 7 (Spampinato et al., 2014), implying a larger number of parameters and the need of evaluating a multiple variable probability $P^{CG}$ , which in turn requires more extensive sampling in the simulation phase. Indeed, the more flexible the functional form, the nearer the final $U^{CG}$ will be to the “true” U : if all the correlations were included, one would have $U^{CG}\left(\left\{Q,\gamma \right\}\right)\equiv -k_{B}T\ln\left(P\left(\left\{R\right\}\right)\right)=U\left(\left\{R\right\}\right)+const$ , at the first iteration. On the other hand, one can show that, with a sufficiently flexible potential, even the REM method converges exactly to the many-body PMF (Chaimovich and Shell, 2011). Therefore, in principle, the two methods are equivalent, and both include the entropic part of the PMF, although in practice, using different numerical procedures, they may lead to different numerical results.

Focusing on the forces rather than on probabilities, the force matching (FM) method minimizes the following loss function (Equation 10):

$$L_{\text{FM}}={\left\langle \underset{I}{\sum }|F_{I}\left(R\left(r\right),\gamma \right)-f_{I}\left(r\right){|}^{2}\right\rangle }_{\text{A}A}\;\;F_{I}\left(R,\gamma \right)=-\nabla_{R_{I}}U^{CG}\left(R,\gamma \right)\;f_{I}\left(r\right)=\underset{i\in I}{\sum }f_{i}\left(r\right),$$ (10)

that is, the mean square difference between the CG forces $F_{I}$ derived from the CG potential, and the atomistic forces $f_{I}$ , defined for each bead I as the sum of the forces acting on all atoms i mapping to that bead. The minimization is performed over configurations R(r) obtained by mapping the atomistic coordinates, so that CG and atomistic forces are always evaluated on corresponding configurations. Although it explicitly targets mechanical consistency rather than thermodynamic consistency, this method has been proven equivalent to REM and IBI since it reproduces the multidimensional PMF (including its entropic part), provided that the sampling is statistically adequate and the potential form is sufficiently flexible (Noid et al., 2008). As with REM and IBI, this equivalence is not guaranteed when using simplified or decoupled functional forms (Chaimovich and Shell, 2011); however, it was shown (Noid et al., 2007) that FM can effectively capture the higher-order correlations better than REM. A comparative summary of the features of these methods is reported in Table 1.

**TABLE 1:** Comparative table of the methods to parameterize analytical coarse-grained potentials $U^{CG}\left(Q,\gamma \right)$ .

Method	IBI	REM	FM	Free energy (PMF)
**Target quantity or loss function**	Marginal distributions $P_{i}\left(Q_{i}\right)$	$L_{\mathrm{REM}}^{i}={\left\langle \frac{\partial U^{CG}}{\partial \gamma_{i}}\right\rangle }_{\text{AA}}-{\left\langle \frac{\partial U^{CG}}{\partial \gamma_{i}}\right\rangle }_{\text{CG}}$	$L_{FM}=\left\langle \sum_{I}|F_{I}^{CG}-f_{I}^{AA}{|}^{2}\right\rangle$	$L_{\mathrm{PMF}}={\left(U^{CG}-\left[\bar{u}-T\Delta S^{CG}\right]\right)}^{2}$
**Correlation treatment**	Usually only implicit via single variable marginal distributions	Explicit correlation terms can more easily be included	Implicit 3-body captured in pairwise form	Explicit correlation terms can be included
**Input data**	Structure dataset (from any source)	Structure dataset (from any source)	Atomistic structure and force datasets	Atomistic structure and energy datasets
**Ensemble averages**	Dataset average +average over CG simulations at each iteration	Dataset average +average over CG simulations at each step	Average over the atomistic dataset (upon mapping of atomistic forces onto CG)	Average over the atomistic dataset (upon average over CG macrostate)
**Computational cost**	High	High	Moderate	Low–moderate
**Sensitivity to dataset sampling**	Moderate: undersampled regions affect $P^{i}\left(Q_{i}\right)$ , iteratively corrected	Low: relative populations corrected at each step via ensemble comparison	High: transition states must be well sampled in the dataset	Low: free energy is directly matched
**Numerical stability**	Low: due to unaccounted coupling between $P^{i}\left(Q_{i}\right)$ s	High: especially when explicit correlations are included	Low: noisy due to mapping and force derivation	Low–moderate: noisy due to mapping

These approaches face fundamental limitations especially when applied to disordered proteins since the simple decoupled form fails to capture the cooperative nature of conformational changes that may drive aggregation. In fact, these proteins are characterized by complex, multi-basin energy landscapes with small barriers separating compact non-aggregation-prone states from extended β -sheet-rich aggregation-prone conformations. Taking A β 42 as a concrete example, this protein can populate an ensemble of interconverting heterogeneous states, including β -hairpin motifs, fully disordered coils, or transient helical structures (Bini et al., 2026). The problem is further worsened by the difficult treatment of hydrogen bonding in CG models, strongly correlated with conformational terms, yet extremely relevant in determining both internal conformation and aggregation (Iadanza et al., 2018). To describe these features, statistical methods such as REM perform better than FM (Noid, 2023), more accurately returning the relative populations of different conformational states and barriers (Thaler et al., 2022), while FM is overly sensitive to the dataset, leading to inaccurate estimation of undersampled regions, such as barriers. In general, however, the issue of poor correlations and undersampling are connected: the former could be mitigated by adding explicit correlation terms to the functional form, but this requires a more extensive sampling, increasing the computational burden and worsening the instability and possible underdetermination of the parameterization procedure.

## 3 AI-based potentials and force fields

In summary, the main limitations in the standard CG approaches stem from the poor treatment of correlations among the explicit degrees of freedom, which in turn may depend on oversimplified functional forms and/or instabilities in the parameterization procedures. AI methods can overcome these limitations and offer solutions at different levels: they can improve the parameter optimization procedure of a given potential form, or they can deliver transformative potentials with high levels of transferability (Majewski et al., 2023).

The implementation of more complex functional forms for the CG potentials is a commonly used approach especially to describe the hydrogen bonds, needing terms correlated with conformational variables (Alemani et al., 2009; Yap et al., 2008), the side chain interactions needing anisotropic representations (Liwo et al., 2019; Alemani et al., 2009), and even electrostatics, where the bare Coulomb form is insufficient to represent the ionic screening (Spiga et al., 2013; Bini et al., 2023). These forms preserve a physical meaning, which allows the analytical post-processing of the results. Pushing the approach to the extreme, one could abandon the separated parametric functional form, and let the complete many-body potential be the subject of the optimization, loosing in this case the direct physical intepretability. As mentioned, however, as the number of parameters increases, the fitting procedure becomes increasingly complex and requires larger amount of data. This is where AI methods can provide support as they are particularly suited to optimization tasks in which correlations dominate.

The general problem can be recast in a form suited for ML-based optimization: given a dataset of conformations R associated to loss function L({R},{γ}) depending on them and on a set of parameters {γ} , find the set of parameters that minimize L . Different approaches differ by the content and form of L and model architecture, which in turn can influence the efficiency, accuracy, and interpretability of the model. In the following section, we examine these approaches systematically.

### 3.1 Loss functions

The concept of loss function has already been introduced in the previous section as it is also used in the traditional parameterization methods, but it is a more general and flexible concept which can be adapted to different needs.

The **force-based loss function** $L_{FM}$ is defined in Equation 10. It requires as input a dataset of configurations and forces usually from atomistic simulations and targets directly the CG forces (Wang et al., 2019; Wang et al., 2020 J.). As mentioned, its weakness is the under-sampling of barriers (Thaler et al., 2022) and sensitivity to numerical noise, which is especially apparent in the evaluation of energy derivatives and is amplified by the operation of mapping of the atomistic forces on the CG forces (Wang et al., 2019). The cost is mainly due to the need of handling forces in addition to structures, but the statistical average is performed only on the input atomistic dataset.

The **entropy-based loss function** $L_{\mathrm{REM}}$ is defined in Equation 8 and targets the minimization of $S_{\text{rel}}$ through the differences in its gradients with respect to parameters. As mentioned, the computational cost is higher than that in FM-based methods, needing averages over CG configurations and therefore CG simulations at each step in the parameter space, but it generally results in more accurate free energy surfaces and barrier heights (Thaler et al., 2022).

The **free-energy-based loss functions** $L_{\mathrm{PMF}}$ share with the $L_{FM}$ the use of the atomistic dataset only for statistical averages but directly target energies instead of forces, thereby avoiding the calculation of gradients. The method is widely used especially to develop empirical atomistic force fields from *ab initio* data (Kovács et al., 2023), where the targets are the potential energy surface (PES). To extend the method to the CG PMF, that is a free energy surface (FES), one must explicitly account for the entropic contribution. The loss function is therefore defined as the difference between the CG potential and the atomistic mean energy $\bar{u}\left(R\right)$ corrected by the entropic contribution $T\Delta S^{CG}$ , as shown in Equation 11:

$$L_{\mathrm{PMF}}=\underset{R}{\sum }{\left(U^{\text{CG}}\left(\gamma ,R\right)-\left[\bar{u}\left(R\left(r\right)\right)-T\Delta S^{CG}\left(R\right)\right]\right)}^{2},$$ (11)

where the summation is over the CG configurations obtained from mapping those in the atomistic dataset. $\Delta S^{CG}=S_{\mathrm{rel}}^{CG}+S_{\mathrm{map}}$ includes all entropic terms derived by the probability coarse graining and mapping (see Equation 3). The average over the atomistic dataset is implied within the mean $\bar{u}\left(R\left(r\right)\right)$ . $S_{\mathrm{map}}\left(R\right)$ includes the microscopic degeneracy of R , and the relative entropy depends on the fluctuations of u(R) within the subset of the same microstates, which can be evaluated as $S_{\mathrm{rel}}^{CG}\left(R\right)=\left(k_{B}\beta^{2}/2\right)\bar{{\left(u\left(R\left(r\right)\right)-\bar{u}\left(R\left(r\right)\right)\right)}^{2}}$ (Giulini et al., 2020). Being the free energy surface directly targeted, the sensitivity to undersampled regions is reduced, and because the calculation of energy gradients is not involved, the related numerical noise is absent (Table 1).

The **structure-based loss functions** target conformation-dependent observables O evaluated on the atomistic and CG ensembles, weighted by coefficients $w_{k}$ reflecting their relative importance, as shown in Equation 12:

$$L_{\text{struct}}=\underset{k}{\sum }w_{k}{\left(O_{k}^{CG}\left(\gamma \right)-O_{k}^{AA}\right)}^{2}\;\;O_{k}^{AA}={\left\langle o_{k}\left(r\right)\right\rangle }_{AA}\;\;\;O_{k}^{CG}={\left\langle o_{k}\left(R\right)\right\rangle }_{CG,\gamma }$$ (12)

where the average over the atomistic dataset is used as the estimator of $O^{AA}$ , while the evaluation of $O^{CG}$ requires CG trajectories and therefore, as in REM and free-energy-based methods, requires a simulation at each step in the parameter space (Ivanov et al., 2023; Leonarski et al., 2013). The advantage of this kind of L is the possibility of customizing the combination of different observables, such as radius of gyration, end-to-end distances, or other geometrical properties, with chosen weights $w_{i}$ . More in general, combined L s are very useful in the case of disordered proteins because multiple physical observables must be simultaneously matched to capture the aggregation behavior. For example, A β aggregation-prone conformations exhibits an increase in the radius of gyration, and this structural change is coupled to the formation of hydrogen-bonding patterns, strongly impacting the free energy (Bini et al., 2026). Matching structural and energy changes together helps capture the correct conformational transitions. Therefore, a useful form of the loss function could be $L_{\text{total}}=\lambda_{E}L_{\text{energy}+\text{entropy}}+\lambda_{\text{struct}}L_{\text{struct}}$ , where the weights $\lambda_{E}$ and $\lambda_{\text{struct}}$ balance energetic and structural accuracy.

### 3.2 Machine-learning-aided analytical potential optimization

The optimal parameterization stems from the minimization of any of the L s. In the following section, we discuss various ML approaches offering different trade-offs between interpretability, accuracy, and computational cost, also summarized in Table 2. In conventional methods, minimization usually proceeds by regression or descent along the $\nabla_{\gamma }L$ gradient, as shown in Equation 13:

$$\gamma^{\left(t+1\right)}=\gamma^{\left(t\right)}-\eta \nabla_{\gamma }L\left(Q,\gamma^{\left(t\right)}\right),$$ (13)

which represents the iterative update of the parameters γ in the direction that most steeply reduces the loss function, with η controlling the step size of each update. The cost of calculation and possible numerical instabilities stem from a large number of parameters and their correlations. Addressing the simultaneous optimization of all parameters is necessary to account for their correlations, and this is where ML methods can increase efficiency by solving Equation 13 using automatic differentiation. The problem is represented as a computational graph, in which the leaf nodes are the parameters γ and the configurational data Q , and the root node returns L . The chain rule for derivatives is used to split the calculation in connected simple operations and may proceed by forward and backward passages, leading finally to the simultaneous calculation of all the partial derivatives $\partial L/\partial \gamma_{i}$ , at a computational cost proportional to that of a single evaluation of L , independently of the number of parameters (Frank, 2022). The convergence of Equation 13 can be improved by adding a regularization term to the loss function. This is particularly relevant when parameters are physically correlated, such that multiple combinations of γ yield equally valid values of L . In this situation, individual parameters can drift to large unphysical values that mutually compensate. A regularization term can prevent this by selecting, among all equally valid solutions, the solution with the smallest parameter norm. A practical and robust implementation of this regularized gradient descent is provided by the Adam optimizer (Kingma and Ba, 2015). This approach has been used in various contexts and with different types of L : in lipid CG models, targeting the mixing density (Carrer et al., 2024); for disordered proteins, targeting radial distribution function Greener 2024); and even to optimize atomistic FFs using energy-based L derived from quantum calculations (Wang et al., 2023). Especially, if used with L s that do not require CG simulations, this method can be relatively fast, scaling with the number of parameters and the size of the input dataset ( $N_{\mathrm{params}}\times N_{\mathrm{conf}}$ , see Table 2 column 1), and therefore requiring limited resources. The appropriate size of the input dataset for training is generally $∼10\times N_{\mathrm{params}}$ , which is easily obtainable from atomistic simulations or experiments.

**TABLE 2:** Comparison of ML approaches for CG-FF optimization. E = number of training epochs; d = descriptor vector dimension (kernel methods); D = depth (number of hidden layers), W = width (neurons per layer in NN); F = number of input features or feature dimension per node (graph NN); $n_{\text{bins}}$ = number of histogram bins for distributions; N = number of CG beads. Number of parameters and related dataset size are those of a typical CG protein model.

Method	Loss function minimization	Reverse probability mapping	Kernel expansion	Fully connected NN	Graph NN + dense NN
**Model representation**	Analytical FF: U(R;γ)	Analytical FF: U(R;γ)	$F\left(R\right)=\sum_{i}\alpha_{i}\;\nabla_{\;R}\;k_{U}\left(R,R_{i}\right)$	$U=U_{\text{prior}}+U_{\text{NN}}\left(R;W\right)$	$U=U_{\text{prior}}+U_{\text{GNN}+\text{NN}}\left(R;W\right)$
**Core calculation**	Automatic differentiation of L	NN-based reverse mapping of P(γ)γ=NN(P(γ))	Kernel matrix inversion	Backpropagation; “CGnet” architecture	Backpropagation; “SchNet” message-passing + CGnet architecture
**Learned parameters** $N_{params}$	100–500 FF parameters	$10^{3}$ – $10^{4}$ NN weights	$10^{4}$ – $10^{5}$ expansion coefficients $\alpha_{i}$	$10^{5}$ – $10^{6}$ NN weights	$10^{5}$ – $10^{6}$ NN weights
**Dataset size** $N_{ocnf}$	$10^{3}$ – $10^{4}$	$10^{4}$ – $10^{5}$	$10^{3}$ – $10^{4}$	$10^{6}$ – $10^{8}$	$10^{6}$ – $10^{8}$
**Training complexity**	$O\left(E\cdot N_{\text{params}}\cdot N_{\text{conf}}\right)$	$O\;\left(E\cdot N_{\text{conf}}\cdot \left(n_{\text{bins}}\cdot W+D\cdot W^{2}\right)\right)$	$O\left(N_{\mathrm{conf}}^{3}\right)$	$O\;\left(E\cdot N_{\text{conf}}\cdot \left(F\cdot W+D\cdot W^{2}\right)\right)$	$O\;\left(E\cdot N_{\text{conf}}\cdot D\cdot \left(N^{2}\cdot F+N\cdot F^{2}\right)\right)$
**Small data performance**	Good	Moderate	Excellent	Poor	Poor
**Large data performance**	Good	Good	Poor	Excellent	Excellent
**Simulation cost**	Fast (analytical)	Fast (analytical)	$O\left(N_{\mathrm{conf}}\cdot d\right)$	$O\left(F\cdot W+D\cdot W^{2}\right)$	$O\;\left(D\cdot \left(N^{2}\cdot F+N\cdot F^{2}\right)\right)$
**Active learning**	✗	✗	✓	✗	✗
**Interpretability**	✓	✓	✗	✗	✗
**Predictivity and accuracy**	Moderate	Moderate–Good	Good	Excellent	Excellent
**Typical application scenario**	Small dataset, large-scale aggregation mechanism	Moderate dataset, large-scale aggregation mechanism	Small dataset, complex multi-basin energy landscape	Large dataset, accurate FES of small protein or dimers	Large dataset, study across structurally diverse protein families

Changing the perspective, an alternative approach trains a NN to perform the inverse mapping from target distributions P(Q) evaluated over the input dataset back to the γ s of the given functional form. The NN is trained over a dataset of CG simulations performed with CG-FF with different {γ} to find the relationship $\gamma ↔P^{CG}\left(Q,\gamma \right)$ and subsequently used to find the set of parameters that return the target distributions P(Q) , i.e., γ=NN(P(Q)) . The simplest implementation of this method targets single variable distributions, as applied, for instance, to polymers (Shireen et al., 2022) or molecular liquids CG FFs (Moradzadeh and Aluru, 2019) for the bonded terms or Lennard–Jones parameters. As the IBI, this approach could fail to catch correlations among variables. To solve this issue, one may consider using joint distributions as input, in the first instance only two variable distributions (e.g., for angles and dihedrals and hydrogen bonding parameters), and train the NN such as $\left(\theta ,\phi ,\gamma_{nb}\right)=\text{NN}\left(P\left(\theta ,\phi \right),P\left(\phi ,\gamma_{hb}\right),\ldots \right)$ . This would improve the representation of correlations particularly relevant in given problem, such as those between backbone conformational terms and hydrogen bonds in disordered proteins (Tozzini et al., 2006). Particularly, in A β , the interconversion between compact states and extended states depends on the formation of hydrogen bonds, making their joint treatment necessary.

In this method, the trained parameters are those of the neural network, i.e., weights of the edges and biases of the nodes. For a fully connected network with D hidden layers, each of width W (number of nodes per layer), these scale as $W^{2}\cdot D$ . According to Shireen et al. (2022), a NN with W∼80 and D∼4 (i.e., approximately $W^{2}\cdot D∼10^{3}$ – $10^{4}$ ) is used to back-map six FF parameters, using a dataset of ∼ 5,000 configurations (Table 2, column 2). One may therefore infer that backmapping ∼ 100 parameters of a typical of a CG FF would require approximately $10^{5}$ configurations in the dataset, significantly larger than what is typically needed for direct gradient-descent optimization.^5^ The training cost is $E\cdot N_{\text{conf}}\cdot \left(n_{\text{bins}}\cdot W+D\cdot W^{2}\right)$ , where $n_{\text{bins}}$ is the number of histogram bins used to discretize input distributions (typically 50–100 per distribution) and E is the number of training epochs.

The advantage gained from this larger data requirement is that the method is intrinsically nonlinear, allowing more efficient exploration of the parameter space, whereas gradient descent may converge to local minima. In both of these methods, the functional form of the potential is analytical, and consequently, the cost of its use in CG simulation is small, and its interpretability is high. The other face of the coin of the fixed analytical forms is intrinsically linked to their simplicity: if the chosen functions lack sufficient flexibility, they may not be able to capture the complex, multi-basin energy landscape characteristic of disordered protein systems.

### 3.3 Kernel methods

A more flexible alternative is provided by kernel methods, in which the potential U has not a definite form; rather, it is treated as a set of Gaussian variables with expected average $\mu =\mu_{U}\left(R\right)$ and expected covariance $K_{ij}=k_{U}\left(R_{i},R_{j}\right)$ and therefore distributed according to $p\left(u\right)\propto \exp\left(-\frac{1}{2}{\left(u-\mu \right)}^{⊤}K^{-1}\left(u-\mu \right)\right)$ . This is named the Gaussian process and denoted as $U\;∼\;GP\;\left[\mu_{U}\left(R\right),\;k_{U}\left(R,R^{\prime }\right)\right]$ . Because forces are gradients of potentials and differentiation is a linear operation, it turns out that also forces are a GP, with mean $-\nabla \mu_{U}\left(R\right)$ and covariance $k_{f}\left(R,R^{\prime }\right)=\nabla_{R}\;k_{U}\left(R,R^{\prime }\right)\;\nabla_{R^{\prime }}^{⊤}$ . In the gradient-domain machine learning (GDML) approach, the kernel matrix $k_{f}$ evaluated over a training dataset of structures and forces is inverted to obtain the forces on new configurations. The final force prediction formula is presented in Equation 14:

$$F\left(R\right)=\overset{N_{\mathrm{conf}}}{\underset{i=1}{\sum }}\alpha_{i}\;\nabla_{R}\;k_{U}\left(R,R_{i}\right),$$ (14)

where $\alpha_{i}$ is the learned coefficient of the inverse matrix.

Previously used to build empirical atomistic FFs from quantum mechanical calculations (Deringer et al., 2021), these methods were recently applied to CG models of an alanine dipeptide (Wang et al., 2020a). The resulting variance of the U(R) distribution quantifies the uncertainty of the prediction, which naturally increases in regions of configurational space poorly sampled by the training data. This can be used to guide the selection of new training configurations iteratively improving the model, also known as active learning, and allows augmenting data without reducing the information content, thereby achieving higher accuracy. GDML can achieve high accuracy with an amount of input conformation comparable with that of classical regression approaches (Wang et al., 2020a). In general, the total number of learned parameters scales as $N_{\mathrm{conf}}\times \left(d+3N_{\text{beads}}\right)∼N_{\mathrm{conf}}\times N_{\mathrm{beads}}^{2}$ (when descriptor d is the inter-bead distance) reaching $∼10^{5}$ for systems as small as chignolin with $N_{\mathrm{conf}}∼10^{3}$ (see Table 2 column 4).

The primary limitation of kernel methods is the computational cost. The training complexity scales as $O\left(N_{\mathrm{conf}}^{3}\right)$ mainly because the inversion of the kernel matrix soon becomes prohibitive. In addition, the force evaluation during MD simulations requires computing the kernel $k_{U}\left(R,R_{i}\right)$ for all $N_{\mathrm{conf}}$ stored training configurations at each timestep, resulting in a cost of $O\left(N_{\mathrm{conf}}\times d\right)$ per evaluation, typically $10^{4}$ – $10^{5}$ operations. This overhead limits the simulation speedup achievable through coarse-graining. On the other hand, at variance with predetermined functional forms, they can more accurately reproduce a complex energy landscape through their weighted combination of kernel basis functions, enabling representation of multimodal free energy surfaces characteristic of intrinsically disordered proteins (Cyzova et al., 2023).

### 3.4 Generalities of neural network force fields

One extreme approach to overcome the limitations of fixed functional forms is directly using a neural network as the force field, with forces represented in Equation 15:

$$F\left(R\right)=\text{NN}\left(R;\theta \right),$$ (15)

where {θ} denotes the network weights and biases optimized during training, which take the role of the FF parameters. In several current implementations, the NN learns forces from molecular configurations through a hierarchical process: it extracts geometric features (e.g., distances, angles, and dihedral) from each configuration R and uses them as input for learning. These features pass through D hidden layers with W neurons, each connected to every neuron of the previous layer (dense NN), and the final output layer predicts forces on the CG beads. In principle, full nonlinear relationships between structure and forces can be included in a NN FF, which can mimic the actual many-body interactions. The predicted forces are then used to drive MD simulations, generating trajectories that can be analyzed to reconstruct the complete energy landscape.

In principle, this approach can reproduce the multi-basin landscapes of disordered proteins—features without the limitations of the fixed functional forms, but it has two types of drawbacks. On the practical level, both the computational cost of simulation and that of training are increased, the latter even because the dataset needed for the proper training is very large. The cost to perform simulations is generally increased from 10- to 100-fold with respect to analytic FF representations (Unke et al., 2021; Wang et al., 2021). Second, the lack of terms with explicit physical meaning in the FF decreases controllability and interpretability of the result.

### 3.5 Physics-informed neural network force fields

The need of mitigating the drawbacks of NN-FF has motivated the development of physics-informed approaches that combine the flexibility of neural networks with physical constraints. For instance, CGNet (Noé et al., 2020; Wang et al., 2019) is an architecture specifically designed for CG models, which decomposes the potential in two terms, as shown in Equation 16:

$$U_{\text{CG}}\left(R\right)=U_{\text{prior}}\left(R;\gamma_{\text{prior}}\right)+U_{\text{NN}}\left(R;W\right),$$ (16)

where $U_{\text{prior}}$ includes physical information through explicit analytical restrains (e.g., repulsive terms to avoid interpenetration of beads) to prevent the formation of forbidden conformations, while $U_{\text{NN}}$ captures complex components (e.g., non-bonded interactions and hydrogen bonding networks). CGNet architecture also encodes roto-translational and permutational invariance, as well as energy conservation, and has been applied to small proteins such as alanine dipeptide and chignolin, correctly reproducing all the main free-energy minima.

However, the computational cost of the NN-FF scales dramatically with system size. The trainable parameters are all network weights and biases, with the total count depending on the number of structural features F (which increases with system size), the network width W , and depth D as $F\cdot W+D\cdot W^{2}$ ^6^. For instance, alanine dipeptide (5 CG beads and 15 geometric features) requires $∼10^{5}$ parameters (for W=160 , D=5 ), while small proteins such as chignolin require $∼3\times 10^{5}$ parameters (for W=250 , D=5 ). Therefore, training requires datasets of $10^{6}-10^{8}$ input configurations (Table 2 column 6), which is several orders of magnitude larger than those required by regression approaches. The training complexity scales as $O\left(N_{\mathrm{epochs}}\cdot N_{\mathrm{conf}}\cdot C_{\mathrm{forward}}\right)$ , where $N_{\mathrm{conf}}$ is the dataset size and $C_{\mathrm{forward}}=O\left(F\cdot W+D\cdot W^{2}\right)$ represents the cost for a single forward pass, corresponding to the energy/force computation for a single configuration. Most critically, during MD simulations, a forward pass is needed at every timestep, introducing an additional overhead of ∼ 10–100 times compared to analytical FFs, potentially limiting the speedup achievable through coarse-graining.

In a subsequent version, CGnet learns the *N*th body interactions separately (up to fifth) using the form $U_{NN}=\sum_{k=2}^{5}U_{\text{NN}}^{\left(k\right)}\left(R;W_{k}\right)$ , to determine which order of approximation correctly reproduces the free-energy landscape. The learning proceeds hierarchically: first, the two-body potential $U_{NN}^{\left(2\right)}$ is learned by associating the $\left(\begin{array}{c} n\\ 2 \end{array}\right)$ pairwise input distance $r_{ij}$ ( n= the number of CG beads) to network units of sufficient size and outputs the corresponding two-body energy contributions. Subsequently, $U_{NN}^{\left(2\right)}$ is fixed, and the three-body correction $U_{\text{NN}}^{\left(3\right)}$ is learned using $\left(\begin{array}{c} n\\ 3 \end{array}\right)$ units (one for each of the three pairwise distances among a triplet {i,j,k} ), and so on, at each order k , $\left(\begin{array}{c} n\\ k \end{array}\right)$ network units compute k -body energy contributions (Wang et al., 2021). The number of network units scales combinatorially with system size, and each of them consists of W∼60 –80 and D∼3 , approximately 3–4 times smaller in width than the whole uncoupled CGnet ( W=250 , D=5 ), and it was shown that it can bring a spare in training cost. In addition, this methodology allows analyzing the multi-body order needed for an accurate representation of the free energy, which, in the case for chignolin, turned out the fifth. In fact, the key advantage of the multi-body decomposition is its physical interpretability: unlike the original CGnet, where all interaction orders are entangled in a single dense network, the multi-body expansion explicitly separates the contributions of each nth-body order, allowing to assess their relative importance in shaping the energy landscape.

In addition to CGnet, which we used as an example of physics-informed NN, similar approaches include, among others, DeepCG (Zhang et al., 2018) and Allegro/DeePMD (Loose et al., 2023), which incorporate to different extent system symmetries as physical constraints. In general, making the restraints less specific or weaker improves the transferability and accuracy of the NN-FF but increases the cost and reduces explainability.

### 3.6 Graph neural network force fields

In the NN-FF approach, selected features (e.g., internal variable sets) are extracted from a learning dataset and used to train the NN to predict forces. SchNet (Husic et al., 2020), originally developed for atomistic systems, introduces a key architectural innovation: molecular systems are directly represented as graphs (G-NN) where features are learned rather than predetermined. SchNet is used to predict energies and forces for dynamics, but GNNs have more general capabilities and were recently also proposed to directly predict trajectory propagation (Zheng et al., 2020; Sanchez-Gonzalez et al., 2020; Immanuel et al., 2026; Guo et al., 2023), learn specific collective variables that capture slow dynamics, or enable the automatic identification of relevant conformational states or hidden patterns during dynamics (Ahsan et al., 2026).

In GNN, each CG bead becomes a node of the graph, described by a feature vector, which is connected by edges to other beads. Each node is initialized with a small set of features encoding the type identity (e.g., amino acid type), while edges encode the inter-bead distance. During training, at each step, the node features are updated based on the features of its neighbors, iterating through D message-passing layers. At variance with standard dense NNs, each bead receives and passes information (messages), with its neighbors accumulating structural context from beads up to D steps away in the molecular graph. After a sufficient number of interactions, each bead has acquired its own identity, the distances to all neighboring beads, and the many-body context, resulting in a vector of dimension F (typically $10^{2}$ ). This representation with learned features is then used as input for another dense NN to predict energy/forces/dynamics.

For a given size of the training dataset, this approach displays improved accuracy in the free-energy surface reproduction for chignolin (Husic et al., 2020) and has shown transferability to larger and structurally diverse proteins of average size (up to ∼100 residues) (Charron et al., 2025). The improved accuracy is mainly due to the density of the NN, while transferability across different molecular systems is due to the GNN capability of learning the features: although the hand-selected features of CGnet are system-specific (fixed number of distances, angles, and torsions depending on the system size and sequence), in CGSchNet, the features are initialized as trainable embeddings per bead type (e.g., amino acid identity), which are shared across different systems. Since these embeddings and the message-passing weights are not system-specific, the same network architecture can, in principle, be applied to different molecules, requiring only fine-tuning rather than complete retraining from scratch.

TorchMD-Net is a GNN inspired by SchNet, used to build CG models of proteins. In contrast to CGSchNet, which outputs leaned features to a dense NN to predict forces, TorchMD-Net directly predicts potential energy for use in the dynamics (Majewski et al., 2023). The protein, as previously described, is represented as a graph of CG beads. After a series of interaction blocks that perform message-passing updates, an output network contracts the feature vector of nodes to a scalar energy contribution summing up to the total energy. Then, forces are computed as its negative gradient and used to perform simulations. As in previous physics-informed approaches, an analytical prior energy term is added, comprising bonded, repulsive, and dihedral chirality terms, to prevent unphysical conformations. This architecture was used to reproduce the folding dynamics of 12 structurally diverse proteins from a large dataset of atomistic simulations, demonstrating true transferability of a single CG potential across multiple proteins. The improved flexibility and transferability come at the cost of increased computation cost. The number of training parameters scales as $O\left(D\cdot F^{2}\right)$ since each massage passing layer has F×F connections, typically leading to $∼10^{5}$ – $10^{6}$ [ D=3 , F=128 in Husic et al. (2020); Majewski et al. (2023)]. More critically, the forward pass complexity scales as $O\left(D\cdot \left(N^{2}\cdot F+N\cdot F^{2}\right)\right)$ , where the number of beads $N^{2}$ term arises from processing all pairwise interactions in the graph and the $F^{2}$ term from the message aggregation at each node. For chignolin ( N=10 beads, F=128 , D=3 ), this amounts to $∼5\times 10^{5}$ operations per force evaluation, substantially expensive as that of CGNet. This also affects the MD simulation speed.

In principle, GNN also enables the direct learning of dynamics via spatial inductive bias, as in the graph neural simulator by Sanchez-Gonzalez et al. (2020). In practice, however, because the network must handle vectorial variables, this strategy needs more physical restrains (e.g., equivariance) to maintain sufficient consistency and avoid dynamical error accumulation. This task is achieved in different ways using specific algorithms. For instance, EGNN (Satorras et al. 2021) and EGHN (Huang et al., 2023) enforce equivariance, but the inclusion of node-specific features in the edge embeddings breaks the symmetry required by Newton’s third law, while DYNAMI-CAL GRAPHNET (Sharma and Fink, 2026) additionally enforces conservation of momenta. Originally designed for granular systems, it has been evaluated on the near equilibrium dynamics of adenylate kinase, displaying performances comparable or better than other similar dynamical predictor models, such as EGHN or EGNN.

### 3.7 Summary of performances of ML-based approaches for CG models

In conclusion, ML methods applied to the building and simulations of CG models display distinct trade-off between accuracy/transferability and interpretability/cost. We now synthesize the above into practical guidance for method selection, also reported in the last row of Table 2, organized around the key constraints a practitioner typically faces, which are system size, data availability, and the relative priority of interpretability versus accuracy. Dataset size requirements, as well as the training and simulation cost, generally increase when moving from ML-parameterized analytical potential or kernel methods to NN-FFs and GNNs. In particular, analytical potentials are trainable with small datasets and extremely cheap for simulations use. The (G)NN requires large or a huge number of configurations for training, which limits their application, also considering the computational cost needed to generate the input dataset with atomistic simulations for large proteins or for their aggregates. In addition, the calculation of forces and trajectories in simulations requires passages through the network at each timestep. Kernel methods locate half way and can quantify uncertainty stemming from low data statistics, also enabling active learning, thereby guiding the selection of new training configurations in poorly sampled regions of conformational space. This keep limited the starting dataset, particularly necessary since these methods are poorly scalable due to the high cost of both training and simulation. In addition, this counteracts dataset bias which is valuable because, beyond size, the quality of available datasets is often limited: data mainly derived from the PDB or from atomistic simulations are often biased toward ordered, well-folded proteins and specific experimental or simulation conditions, with transient aggregation-prone states, such as those relevant for A β , typically underrepresented or absent. As a consequence, models trained on such datasets tend to generalize poorly beyond the conformational space they were trained on. Finally, NN-FFs or GNNs offer high accuracy at the expenses of interpretability and simulation speed, which currently limits their application to very small proteins.

For aggregations studies, particularly extensive sampling of the intra- and inter-molecular conformational space is needed, which increases the burden both in the learning phase and in the simulation production. As a specific example, simulating early-stage A β 42 aggregation requires multi-protein systems of ∼ 100 monomers in boxes of ∼ 250 Å, over timescales of hundreds of nanoseconds, a regime accessible to regression-parameterized analytical FFs, but currently more challenging for NN-FF, whose simulation cost is 10–100 × higher per timestep (Unke et al., 2021). Regression methods therefore emerge as the most practical choice for multi-protein aggregation studies since their computational efficiency enables the required system sizes and timescales, while their physical interpretability allows systematic investigation of how environmental conditions, such as ionic strength or pH, affect aggregation mechanisms. Conversely, the much larger computational cost of NN-FFs currently restricts their use: even small-sized proteins such as A β 42 pose significant challenges; therefore, applications are currently limited to the exploration of the conformational space of individual proteins, although, in this respect, the continuous and rapid evolution of hardware is likely to provide new opportunities soon.

However, NN-FF can be a valuable complementary and refinement tool to address the main weakness of analytical FFs: the comparison of the free-energy landscapes and conformational ensembles obtained from NN-FF simulations with those from regression-parameterized analytical FFs can allow the identification of rare conformations, and regions which the fixed functional form cannot explore. The multi-body expansion as that of CGnet allows identifying up to which level the correlations are involved in the representation of these regions. In general, combined approaches can enable iterative refinement: the NN model highlights which energy basins or barriers are incorrectly represented, guiding targeted modifications to the classical potential’s functional form or parameter ranges.

Although the increased accuracy of physics-informed or graph-based NNs is fundamental for sampling intermediate rare states that fixed functional forms might miss and that are relevant to correctly reproduce the aggregation process, the lack of interpretability remains a drawback of NNs, which is only somewhat mitigated by the use of multi-body expansion. NNs are substantially black box, which may prevent the investigation of aggregation dependence on environmental conditions (for example, ionic strength) or identify which physical components (hydrophobic, electrostatic, or hydrogen bonding) dominate the process in different situations; therefore, adding *a priori* physical information of any kind helps both training and explainability. Therefore, considering hybrid strategies combining the predictive accuracy of NN with the interpretability and computational efficiency of analytical FFs is a perspective key to enable both accurate representation of complex conformational landscapes and systematic exploration of aggregation mechanisms.

## 4 Direct prediction of aggregate structure

We have shown that ML approaches can enter at different levels in the flow connecting potentials → forces → dynamical trajectories, generally improving the accuracy and predictive power and also bringing into the procedure increasing unexplainability (and cost) as one moves downstream. Focusing on the specific problem of protein folding and aggregation, upstream of the flow, we find analytical CG FF parameterized based on statistical datasets (Tesei and Lindorff-Larsen, 2023; Valdes-Garcia et al., 2023), generally less accurate and transferable, but more transparently revealing the specific mechanisms occurring during the process, since the hydrophobicity and electrostatics steric hindrance are explicitly encoded into different FF terms. This transparency is gradually lost in the methods described in the previous section, compensated by increases in accuracy and transferability.

In this perspective, recently developed AI-based structure prediction methods, such as RoseTTafold (Baek et al., 2021; Krishna et al., 2024), ESMfold (Lin et al., 2023), or AlphaFold (Jumper et al., 2021; Abramson et al., 2024), operate very downstream, bypassing the intermediate steps of force calculations and dynamical trajectory production, as these algorithms are trained to directly predict the folded or aggregated structure from the sequence. This improves the efficiency of the result production: these algorithms are able to return a folded structure of an average-sized protein in minutes. The huge impact of these methods in the field of biomodeling was underscored by the 2024 Nobel Prize for Chemistry (Tozzini and Giulivi, 2025) and triggered several dedicated studies on these algorithm performances (Fang et al., 2023; Manfredi et al., 2026). The accuracy of the results depends on several factors, mainly the statistics of the dataset of learning: for the class of well-folded proteins, accuracy reaches 97% .

On the other hand, accuracy decreases substantially in the prediction of aggregate and multimers (Polonsky et al., 2023), especially those involving disordered proteins (Dao et al., 2025), basically due to their low representation in the learning dataset, which necessitates the use of other methods for structural validation (Zhu et al., 2023). Several strategies were considered (Bryant, 2023), which involve the inclusion of information at different levels, such as self-distilled data from the experiment (Ruepp et al., 2010) possibly combined with phylogenetic information (Drew et al., 2021), as well as data from atomistic simulations to be used to enforce the learning, to include physical restrains or possibly missing information, such as those related to the dynamical flexibility otherwise impossible for the algorithm to learn (Bryant, 2023). A different strategy is to extract, in addition to the final results, intermediate information from the algorithm and use it to correct or enforce the parameterization of analytic models where they fail (Bini et al., 2025), leveraging the best capability of ML methods, which is the search of correlations. These hybrid approaches can either provide enhanced accuracy and predictive power of analytic FFs or enhance the NN-FF explainability, or both, taking best advantage of two antipodal methods.

In the following subsection, we focus on AF, based on a deep NN trained on the protein data bank (PDB) and multiple sequence alignment (MSA) databases. AF refines its predictions by processing iteratively the pairwise distance distributions and the MSA information. The algorithm returns a set of model structures obtained by mapping the pairwise distance probability into 3D conformations. In this last part of this study, we describe one among the mentioned hybrid strategies, i.e., how the intermediate information produced by AF can be used in the parameterization of a CG-FF.

### 4.1 Reverse engineering AF predictions and combination with CG-MD simulations

AlphaFold’s predicted structures are accompanied by two accuracy indicators: the predicted aligned error (PAE), which measures the expected positional error between residue pairs, and the predicted local distance difference test (pLDDT), which indicates the confidence of the prediction of the distances to neighboring residues. These are related to other physical quantities also returned by the algorithm, i.e., the contact probability matrices $p_{ij}$ , which are evaluated integrating the more complete distribution of distances (distograms) of each couple of amino-acids in the system. The $p_{ij}$ s can be related to the inter-residue potential terms of the FF (Bini et al., 2025), while PAE and pLDTT can be related to the level of disorder along the chain, enabling the modulation of structural terms in the CG-FFs (von Bülow et al., 2025) and allowing for the optimization of an FF that better describes both ordered and disordered proteins and their mixture.

We illustrate the approach on two specific cases of aggregation of ordered and disordered proteins. The first case regards the aggregation of proteins belonging to the family of intrinsically fluorescent proteins (FPs) (Bini et al., 2025). In this case, the monomer structure, a well-folded regular β− barrel, is returned with an extremely high accuracy by AF. Therefore, the intra-molecular CG-FF terms can be conveniently described with a Go-like model (Trovato et al., 2013). On the other hand, the representation of inter-molecular interactions is rather critical in this system since the phase diagram of aggregation is particularly sensitive to the interplay between hydrophobicity and electrostatics of residues at the interfaces (Bini et al., 2022). AF can generate structures of FPs m -mers (samples for the wild-type GFP are shown in Figure 2), returning as intermediate output a $\left(m\times n_{\text{res}}\right)\times \left(m\times n_{\text{res}}\right)$ matrix ( n= number of residues in the protein) representing the full pair contact probability $p^{m}$ of the multimer. This matrix is composed of n×n monomer-sized blocks, whose diagonal blocks represent the inter-monomer contact probability used by AF to build the monomer, while the out of diagonal blocks can be averaged to evaluate the contact probability between pairs i,j belonging to different monomers (Equation 17):

$$p_{ij}=\frac{\sum_{k,l=1}^{m}p_{i+k\left(n_{\text{res}}\right),j+l\left(n_{\text{res}}\right)}^{m}}{\sum_{ij}^{n_{\text{res}}}\sum_{k,l=1}^{m}p_{i+k\left(n_{\text{res}}\right),j+l\left(n_{\text{res}}\right)}^{m}},$$ (17)

which is related to the inter-molecular interactions. This $p_{ij}$ (represented in Figure 3) can be regarded as an average probability that amino acid i is in contact with amino acid j of another monomer in the aggregate and summarizes all the possible protein–protein interfaces as predicted by AF into a two-protein contact matrix (Bini et al., 2025). The same operation could be performed over the full pair distribution functions $g_{ij}^{m}\left(r\right)$ also returned by AF, to evaluate a monomer–monomer pair distribution matrix $g_{ij}\left(r\right)$ .

**FIGURE 2:**
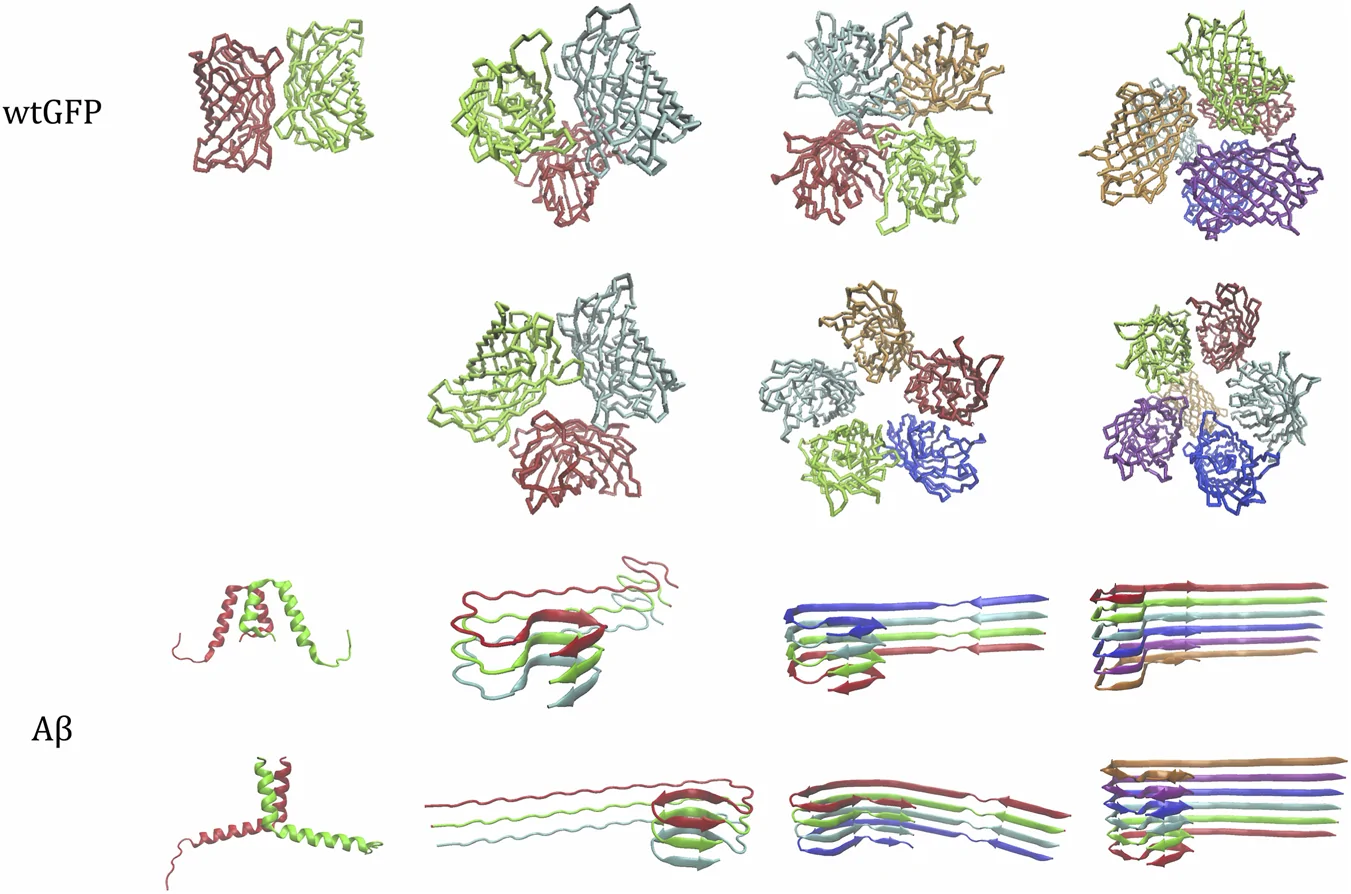
Clusters of increasing size generated by AF3 for the wild-type variant of GFP and the A β protein. For each cluster, the two most structurally diverse predicted structures are reported. In the case of wtGFP, the β -barrel fold is preserved in all clusters, with variability arising only at the inter-monomer interface. For A β , AF3 predicts diverse conformations at the dimer level, while larger oligomers converge toward fibril-like parallel in-register β arrangements.

**FIGURE 3:**
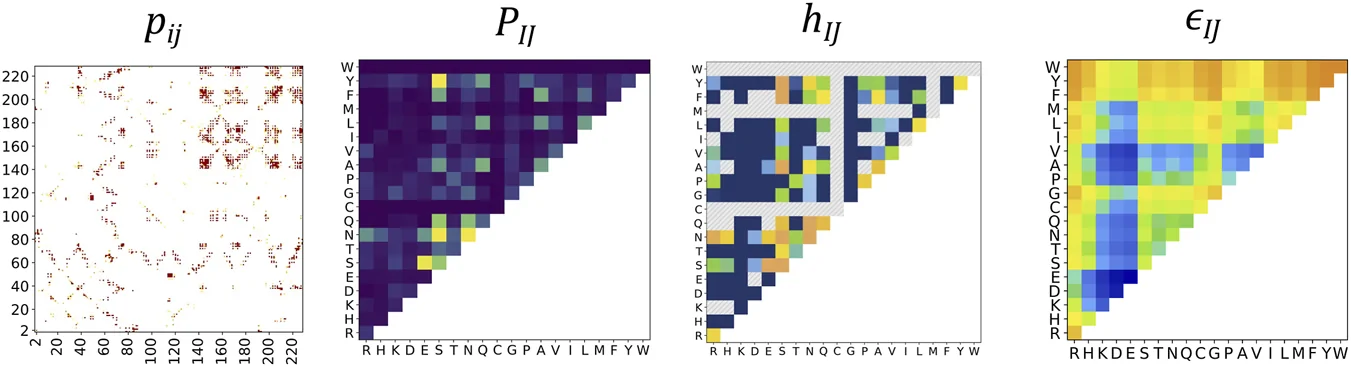
Representation of the matrices $p_{ij}$ (inter-monomer contact probabilities) $P_{IJ}$ (inter-residue contact probability) $h_{IJ}$ (contact energy), obtained as described in the text for the wtGFP, and $\epsilon_{IJ}$ of the CALVADOS FF. Color ranges: for $p_{ij}$ : [0; 0.002] white to red; for $P_{IJ}$ : [0; 0.02] dark blue to yellow; for $h_{IJ}$ : [0; −0.5], blue to orange; for $\varepsilon_{IJ}$ : [0; −0.2] blue to orange; $h_{IJ}=-k_{B}T\ln\left(P_{IJ}/\left(P_{I}^{0}P_{J}^{0}\right)\right)$ with $P_{I\left(J\right)}^{0}$ the average occurrence of amino acid I (or J). Pairs (I,J) are masked in two cases: when one or both amino-acid types are absent from the wtGFP sequence (no structural information available), or when $P_{IJ}<2\%$ of the maximum $P_{IJ}$ value, in which contact signal is below the significance threshold.

In Bini et al. (2025), this information is used to assess the performance of different CG-FF for the FP aggregation, by comparing $p_{ij}$ from AF with those obtained from CG-MD simulations. In principle, however, one may connect $p_{ij}$ with the pair contact probability of amino-acid of types I and J (running along the 20 AA types) with the following relation shown in Equation 18:

$$p_{ij}=\underset{IJ}{\sum }\Pi_{iI}P_{IJ}\Pi_{Jj}^{†}\;\;\;P_{IJ}=\underset{ij}{\sum }\pi_{Ii}p_{ij}\pi_{jJ}^{†},$$ (18)

where the rectangular matrices $\Pi_{iI}$ (and $\pi_{Ii}$ ) transfer the information between the position along the chain and the amino-acid type occupying it, elaborated by a dedicated module of the AF algorithm. In general, they encode key statistical information on the multiple sequence alignment and philogenetics, but restricted to a given protein, $\pi_{Ij}$ is null, except for the element I,j, indicating that location j is occupied by amino acid of type I. Therefore, one may reverse engineer $p_{ij}\to P_{IJ}$ for a specific protein. The result of this operation is reported in Figure 3. Although the $p_{ij}$ matrices highlight the locations of the contact interfaces along the chain, $P_{IJ}$ highlights the amino-acid type more relevant for the interface formation for the specific case (GFP). As expected, $P_{IJ}$ is non-negligible only sporadically since for a given protein, the amino acids relevant for the formation of the interfaces might be specific (in the GFP case, these are, e.g., Glu, Asn, and Ser). Therefore, many of the matrix entries are null. To obtain a more transferable $P_{IJ}$ matrix, the procedure should be repeated for a large subset of different variants within the family, and the resulting matrices should then be averaged. The more general and balanced the set, the more transferable the information. On the other hand, an average performed over a subset of similar proteins (e.g., GFP mutants) would return a $P_{IJ}$ more accurately reproducing the specific interfaces.

If the training was performed on a thermally equilibrated ensemble of structures, $P_{IJ}$ would reflect a Boltzmann distribution. Although this is not always the case, it was previously shown that, even without learning a genuine Boltzmann distribution of states, AF’s learned contact distance distributions can approximate physically relevant energy surfaces (Brown et al., 2023). Note that this assumption has inherent limitations: for instance, PDB structures used for AF training are obtained under heterogeneous experimental conditions, whereas Boltzmann distributions are associated with specific environmental conditions and temperature. However, under this simplifying assumption, $P_{IJ}$ can be related to the potential of mean force via Boltzmann, $P_{IJ}\propto \exp\left(-h_{IJ}/k_{B}T\right)$ , where $h_{IJ}$ is a parameter measuring the average interaction energy per residue pair. In an analytical type potential, this is roughly proportional to the well depth of the $U_{IJ}^{CG}$ potential between amino acids of types I and J, i.e., $\epsilon_{IJ}$ . $h_{IJ}$ is reported in Figure 3 and displays the sparseness inherited from the $P_{IJ}$ due to the poor sequential statistic explored by the GFP sequence. However, when compared to $\epsilon_{IJ}$ of a generic empirical FF for amino acids [we used the CALVADOS (Tesei and Lindorff-Larsen, 2023) for the figure], one can observe that $h_{IJ}$ is sensibly non-null for couples of amino acids interacting more strongly. It is likely that averaging $h_{IJ}$ matrices over a dataset of very diverse proteins, one may converge to a matrix similar to $\epsilon_{IJ}$ , while restricting the average over a family of proteins (e.g., the FPs), one may obtain the set of parameters more accurate for that subset, but less transferable. The same procedure could be applied to the full pair distribution functions $g_{ij}\left(r\right)$ to obtain pair distribution function for AA type and $G_{IJ}\left(r\right)$ -related potentials of mean force $U_{IJ}\left(r\right)∼-k_{b}T\ln\left(G_{IJ}\left(r\right)\right)$ . Therefore, this procedure gives an operative way to extract potential parameters or directly a PMF from AF output, already including a large amount of statistical information learned by the algorithm. The resulting PMF will have a user-defined amount of bias/transferability tuned by the composition of the set of sequences/structures used as input for AF.

This approach is a different example of fruitful combination between ML-based approaches and analytic CG-FF. AF not only captures the system-specific interface but also includes generic statistical information, which can be extracted through the evaluation $h_{IJ}$ and a FF with tunable transferability/accuracy. On the other hand, the comparison between $h_{IJ}$ derived from AF with available FFs allows providing a physico-chemical interpretation to the AF results since the relative weight of hydrophobicity and electrostatics can be directly read from the generated effective FF parameters. On the other hand, the analytic FF can be used in dynamics simulations efficiently exploring the conformational space and re-generating probability matrices $p_{ij}$ or cluster structures to directly compare with AF results. Simulations can dynamically explore even low confidence conformations or aggregate structures, which might be verified *a posteriori* or even used as augmentation of the structural dataset, and possibly mitigate the problem of lower accuracy of AF on the prediction of aggregate structures. This integration with physics-based CG modeling also provides physical interpretability, revealing which specific residue–residue interactions drive aggregation and enabling systematic investigation of how environmental conditions or mutations modulate these interactions.

### 4.2 Hybrid AF–MD approaches for disordered proteins

AF uses multiple sequence alignments, which conceptually exploits the conservation of functional protein structures by evolutionary pressure, and operates assuming Anfinsen’s postulate stating that a sequence uniquely folds in its native structure (Ghosh et al., 2024). The lack of a well-defined structure in the class of multi-stable or disordered proteins already makes the folding problem ill-defined. In addition, the postulate was demonstrated false on these classes of proteins (Sikosek et al., 2016; Nugnes et al., 2026), which is another cause of the poor accuracy of AF for them. These proteins have typically relevant transient conformations not evolutionary conserved. Specifically, the already-mentioned A β disordered proteins exist as a dynamic ensemble of interconverting conformations, leading to the formation of variegate oligomeric intermediate transient species for a single given sequence (Pinheiro et al., 2021).

This issue is worsened by the low statistical representation of these proteins in the conventional structure dataset, which makes the training not sufficient. For instance, specifically considering A β , the PDB database contains only monomer or fibrillar forms of A β (the dominant one), which implies that AF predictions can be biased toward fiber-like conformations and fail to capture the diverse monomeric and oligomeric states that are crucial for understanding the early stages of aggregation. This problem is illustrated by comparing the AF predictions of aggregates of GFP with those for A β (Figure 2). In the case of wtGFP (top), the β barrel fold is conserved in the clusters, and the inter-monomer interfaces are correctly predicted across different variants, so it is the global cluster structure and shape (Bini et al., 2022). By contrast, in the case of A β , AF successfully predicts structurally diverse conformations at the dimer level, while from the trimer on, the predicted structures tend toward fibrillar-like parallel in-register configurations. Although some structurally diverse models, such as helix bundles or β -harpin motifs, are also generated (Santuz et al., 2022), AF provides no thermodynamic weighting between them, making it impossible to assess which conformations are relevant during early aggregation. The regular structure learned from the dataset reinforces inter-chain contacts that match mature fibril structures and fail to capture the conformational heterogeneity of early oligomeric assemblies. In addition, experimental studies reveal certain variability also in the fibril structure (Pinheiro et al., 2021), which is not apparently accounted for by AF. Similarly, low performances are observed in other intrinsically disordered protein aggregate prediction (Dao et al., 2025), confirming the importance of training data composition.

Possible strategies to overcome these problems include the complementation with dynamical simulations, possibly performed with intramolecular potentials parameterized or refined with the approaches that ensure the consistency with AF prediction, such as that illustrated in the previous section. The extraction of statistical information from the intermediate output of AF for parameterizing an FF for dynamics, possibly using the same dataset for learning, would ensure internal consistency in the combination of methods and enrich the information and learning. A similar approach was adopted by Pinheiro et al. (2021) using the PAE and pLDDT outputs to restrain disordered regions and by Brotzakis et al. (2025) in which AF’s predicted inter-residue distance distributions have been added as soft restraints in MD simulations performed with CALVADOS force field, thereby generating conformational ensembles consistent with both AF predictions and experimental SAXS data.

It is therefore apparent that possible strategies to correct the failure of AF and of similar ML-based algorithms for structure prediction on disordered proteins is their combination with molecular dynamics simulations used to enforce the dataset in different forms of active learning. More generally, the methods presented in this study can be combined to study the aggregation of proteins and the more challenging process of disordered protein aggregation. Classical parameterization methods (Section 2.3) retain physical interpretability but suffer from poor treatment of correlations and instabilities in the parameterization procedure. This motivates the use of ML-based approaches (Section 3.2–3.6), which can act at different levels of the parameterization workflow, refining parameters or directly predicting the energy landscape, and of AF (Section 4.1), which bypasses this flow entirely by directly predicting aggregate structures from sequence and, despite minimal interpretability, can be used to inform and improve the accuracy of models. The combination of all these approaches can, therefore, preserve both physical interpretability and predictive accuracy necessary to study the aggregation process, from the formation of early disordered oligomers to mature fibrillar assemblies.

## 5 Conclusion and future direction

AI-driven structure prediction tools have stepped into the modeling and study of proteins, surpassing traditional approaches in contests such as CASP as they can predict folds and aggregates in few minutes. However, they are intrinsically little explainable, returning a set of static structures without a rationale. This problem is especially difficult in situations where the accuracy is low since the poor reliability of the result is not accompanied by an explanation which might allow to mitigate it. Incidentally, this occurs in the system interesting for misfolding/aggregation, that is, with proteins with irregular, uncertain, or disordered fold, and in aggregates.

From an antipodal point of view, the problem of (re-)folding and aggregation can be addressed using molecular dynamics simulations with coarse-grained FFs, which offer a high level of explainability and the possibility of dynamically exploring the conformation and configuration space, while providing complete and rich information, such as aggregation pathways, intermediate states, and structure and composition of the interfaces, thereby revealing the mechanistic determinants of the process. The drawback of this methodology is that the accuracy of the results strongly depends on the flexibility of FF, i.e., its capability of including complex effects deriving from internal variable correlations, which in turn depends on the form chosen for the FF and the strategy of parameterization.

However, in an ideal flow connecting the protein sequence to the aggregate structure, the AI methods can enter at different levels. For instance, they can enter the optimization of the parameters of an FF with a conventional parametric form (ML-aided regression or reverse mapping methods), can help flexibilizing the FF form (kernel-based methods), or the NN themselves can be used as FFs (NNs and GNNs) acting as a predictor of forces or even dynamics. Depending on the level at which the ML algorithm enters in the flow, these methods have increasing flexibility and accuracy (and cost, both in the phase of training and in that of use), but generally decreasing explainability. Therefore, the choice of the most appropriate method also depends on the balance between these two aspects in the final results.

In this respect, the AI structure predictors represent the extreme at which the unexplainability dominates. For this reason, we have illustrated in the second part of this study two methods that integrate them with CG-FF simulations with the aim of creating a compromise between the two opposite approaches. In both cases, the statistical information produced as intermediate results by AF (either the contact probability matrices or the estimators of errors) are used to modulate the parameterization of a CG FF. This allows to correct the dynamical simulations for the statistical knowledge included in the learned algorithm and simultaneously make the two approach compatible between each other. In turn, dynamical simulations, made compatible with ML prediction, can be used to complement the ML results or provide additional data to be used in a strategy of active learning.

We reported this as an example of possible strong integration between methods (ML and CG-FF dynamics), which enforce and complement each other, reciprocally compensating their weaknesses. However, in general, it appears that these combinations are possible and beneficial at several different stages of the sequence-to-structure modeling flow. We therefore argue that the future perspective of AI-based modeling is a stronger and more structural integration with more FF-based dynamical simulations, especially in situations such as the misfolding and aggregation of proteins, where it is compulsory to have a dynamical and mechanistic insight of the on-going process. We conclude with the observation that, together with others, such as the genetic algorithms (Leonarski et al., 2013), machine learning belongs to the class of bio-inspired strategies, all exploiting bio-related concepts such as the evolutionary optimization of the loss function, which mimics the fitness to the environment in biological systems. Therefore, the key to properly combining these methods, choosing an efficient loss function, and optimizing other parameters in this complex combined strategy, perhaps, lies in examining real biological examples that have been optimized by nature over approximately four billion years.

## Footnotes

1

v is undefined classically, while it can be related to the De Broglie length in quantum mechanics: $v={\left(2\pi \hbar /\sqrt{3mk_{B}T}\right)}^{3}$ . We also observe that in all this treatment, we disregarded kinetic part of the energy and the related probability distribution, which also brings a similar factor but with dimension $v_{p}={\left(2\pi \hbar \times \sqrt{3mk_{B}T}\right)}^{3}$ , leading to a complex phase space unit of ${\left(2\pi \hbar \right)}^{2}$ .

2 The Boltzmann factor $f\left(x\right)=e^{-\beta x}$ is convex for β>0 ; Jensen’s inequality for convex functions states $f\left(\bar{u}\right)\leq \bar{f\left(u\right)}$ , i.e., $e^{-\beta \bar{u}}\leq \bar{e^{-\beta u}}$ , which applied here gives the ≳ direction.

3 We remark that there is some ambiguity in the literature on the terms: sometimes, either $S_{\mathrm{rel}}$ or $S_{\mathrm{map}}$ is considered the sum of the two terms.

4 The function f(x)=ln⁡x is concave; Jensen’s inequality for concave functions states ⟨f(x)⟩≤f(⟨x⟩) , i.e., ⟨ln⁡x⟩≤ln⟨x⟩ , which applied here gives −⟨ln⁡x⟩≥−ln⟨x⟩ .

5 However, we note that the effectively necessary $N_{\text{conf}}$ is lower in practice since correlated configurations falling within the same histogram bin contribute redundant information.

6 The total number of trainable parameters scales as $W^{2}\cdot D$ in a network of depth D and width W , and the first layer additionally contributes F⋅W weights since each of the W neurons in the first hidden layer is connected to all F features, which leads to the reported total count.
